# NASCI case of the month: a case of anomalous right coronary artery arising from the pulmonary artery

**DOI:** 10.1007/s10554-024-03091-1

**Published:** 2024-04-10

**Authors:** Nicholas Guys, Emily Lippincott, Luba Frank

**Affiliations:** Department of Radiology and Radiological Sciences, Vanderbilt University Medical Center, Nashville, TN USA

**Keywords:** ARCAPA, Anomalous coronary artery, Congenital

## Abstract

Coronary artery anomalies are rare but potentially fatal abnormalities with occasional striking imaging findings radiologists should recognize.

## Case information

**Fig. 1:**
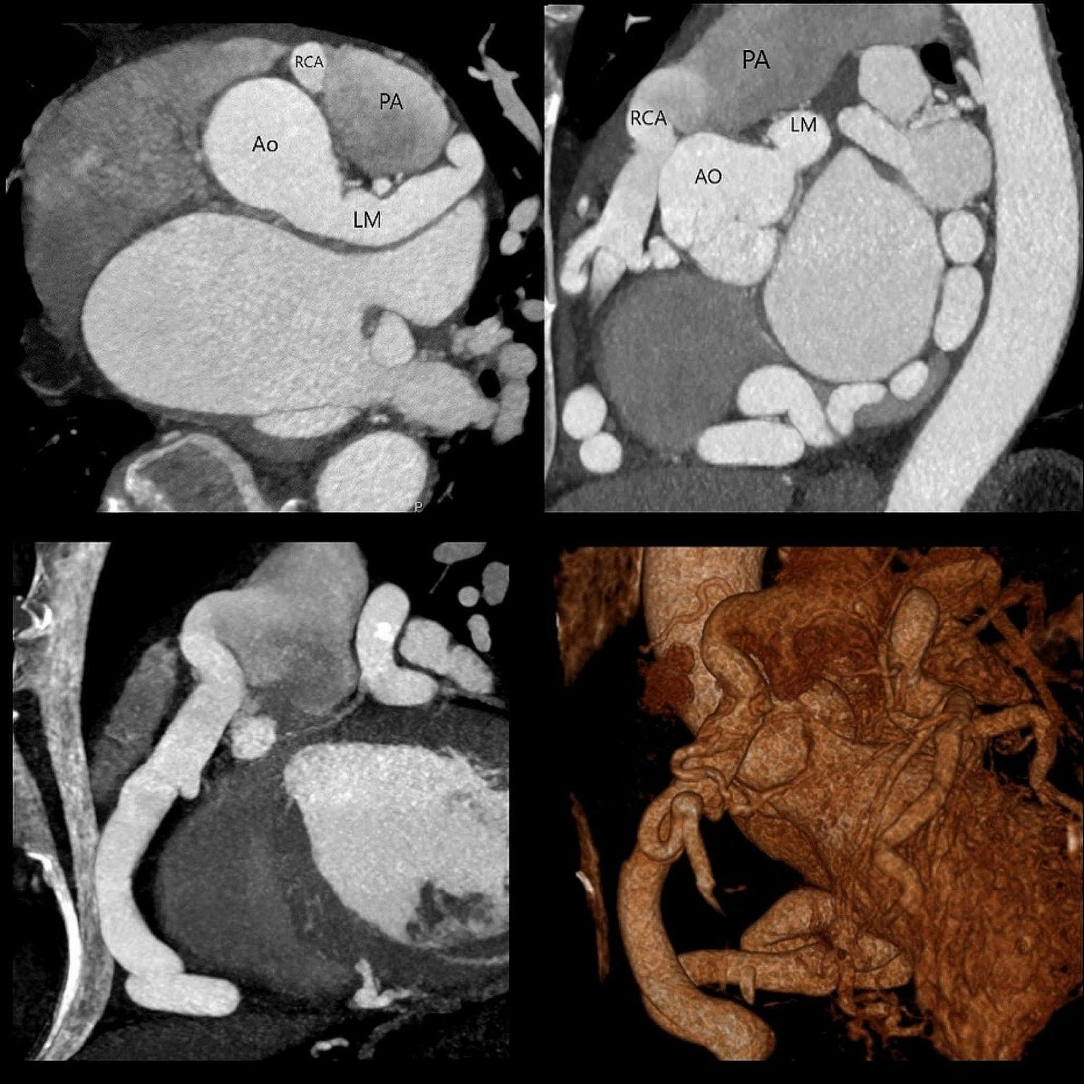
Top Left: An oblique axial plane through the heart and proximal ascending aorta shows dilated left and right coronary arteries. Top Right: An oblique sagittal plane through the heart at the level of the aortic root shows a dilated anomalous right coronary artery arising from the supravalvular pulmonary artery. A dilated, tortuous left coronary system is also shown. Bottom Left: An oblique coronal MIP image shows the dilated anomalous right coronary artery arising from the pulmonary artery. Bottom Right: A 3D vascular rendering shows the anomalous vessel. Ao = aorta, PA = pulmonary artery, RCA = right coronary artery, LM = left main coronary artery.

A 65-year-old female with hyperlipidemia was referred to our department for a calcium scoring cardiac CT. Diffusely dilated right and left coronary arteries were incidentally noted. She subsequently underwent an ECG-gated coronary CTA, which revealed a markedly dilated right coronary artery arising from the supravalvular pulmonary artery as well as severe dilation of the posterolateral ventricular artery and the entire left coronary artery system. Extensive collateralization from the left coronary artery system to the right was noted. She was asymptomatic, with no known cardiac structural abnormalities or other pertinent history (See Fig. 1).

Anomalous origin of the right coronary artery from the pulmonary artery (ARCAPA) is a rare congenital abnormality with an estimated prevalence of two cases per 100,000 individuals [1, 2]. Presentation and physical exam findings are variable but include chest pain, heart failure, myocardial infarction, and murmur with nonspecific ECG findings [1, 2]. Associated cardiac anomalies are frequent, and prognosis is associated with the degree of collateralization with the normal coronary artery [1, 2]. Management depends on clinical factors, though surgical reimplantation is often the treatment of choice [1, 3].

## Data Availability

No datasets were generated or analysed during the current study.
